# Constrictive pericarditis

**DOI:** 10.1007/s10554-026-03603-1

**Published:** 2026-01-19

**Authors:** Aaroh K. Patel, Leila Rezai Gharai

**Affiliations:** 1 1200 E. Marshall Street, Richmond, VA 23298 USA; 2 Virginia Commonwealth University School of Medicine (A.K.P), 1201 E Marshall St #4-100, 23298 Richmond, VA USA; 3 Department of Radiology (L.R.G), Virginia Commonwealth University Health System, 1200 E Marshall St, 23298 Richmond, VA USA

**Keywords:** Constrictive pericarditis, Pericardial calcification, Magnetic resonance imaging, Computed tomography

## Abstract

We report a case of constrictive pericarditis (CP), a rare condition caused by fibrotic pericardial thickening or scarring resulting in restricted ventricular filling. Echocardiography, computed tomography (CT), and cardiac magnetic resonance imaging (MRI) are key in diagnosis.

## Main body

**Fig. 1:**
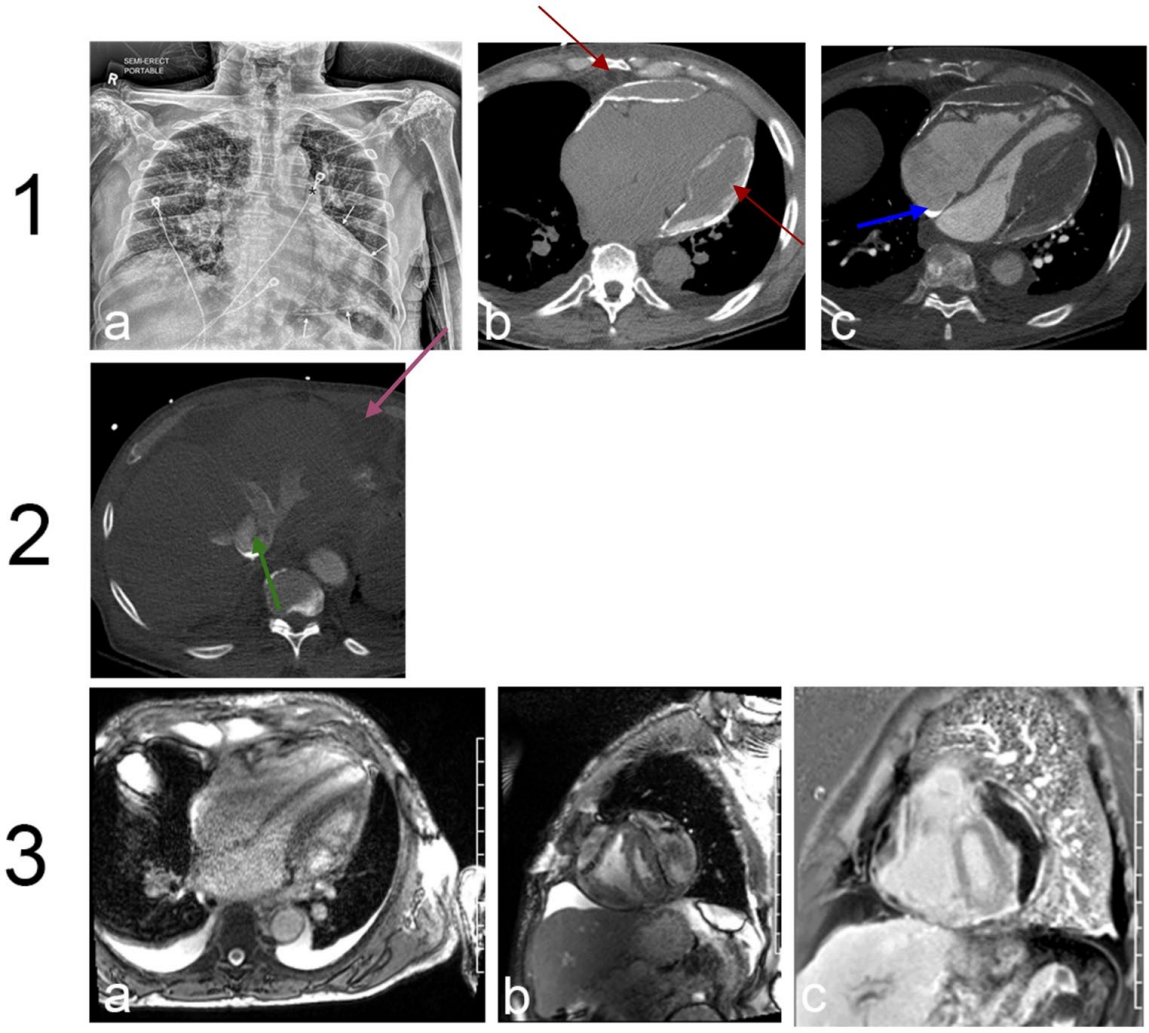
[1]: Chest radiograph depicting cardiomegaly (white arrows) (a). CT angiography in axial view showing dense calcification involving both the visceral and parietal pericardial layers (red arrows) (b). A moderate-sized, loculated pericardial effusion with internal calcific density is seen (blue arrow) (c) [2]: CT angiography in axial view depicting reflux of contrast (green arrows) into the inferior vena cava. Also depicted is hepatomegaly with heterogenous “nutmeg” appearance of the liver parenchyma and predominantly simple-fluid density ascites (pink arrows) (a, b) [3]: Cinematic views of cardiac MRI showing compression of the LV cavity by a thickened and fluid filled pericardial sac and septal bounce (a, b). DGE sequence demonstrates pericardial enhancement (c)

An 80 year-old male was admitted for right facial droop and aphasia. During stroke evaluation, transthoracic echocardiogram revealed a large effusion along the lateral left ventricular (LV) wall. CT angiography was requested to assess cardiac morphology and pericardial effusion volume. Imaging depicted circumferential dense pericardial calcification and partially calcified large-volume pericardial contents (Fig. 1a, b). Ancillary findings included ventricular tubulation (Fig. 1c), bi-atrial enlargement, reflux of contrast into the inferior vena cava, congestive hepatopathy and ascites (Fig. 1[2b]) suggesting restrictive ventricular physiology and right-heart failure. Cardiac MRI obtained for assessment of cardiac function showed severely reduced cardiac output (Fig. 1[3a]), interventricular septal bounce (Fig. 1[3b]) and pericardial enhancement (Fig. 1[3c]). These findings correlated with invasively measured parameters via cardiac catheterization and transesophageal echocardiography. Surgical intervention with creation of a pericardial window was pursued. Operative findings confirmed a densely calcified pericardium, establishing the diagnosis of CP. Tissue culture demonstrated gram-positive cocci. The patient was initiated on intravenous daptomycin and transitioned to oral linezolid with significant clinical improvement.

Etiologies of CP include inflammatory or infectious processes, prior cardiac surgery, mediastinal radiation and idiopathic diseases. Definitive treatment is pericardiotomy/pericardiectomy. Medical management may be pursued in select cases. Our case demonstrates exuberant pericardial calcification and radiologic signs of constriction because of bacterial infection of pericardium.

## Data Availability

No datasets were generated or analysed during the current study.
